# A Systematic Review and Meta-Analysis: Volatile Organic Compound Analysis in the Detection of Hepatobiliary and Pancreatic Cancers

**DOI:** 10.3390/cancers15082308

**Published:** 2023-04-14

**Authors:** Melina Pelling, Subashini Chandrapalan, Emily West, Ramesh P. Arasaradnam

**Affiliations:** 1 Warwick Medical School, University of Warwick, Coventry CV4 7AL, UK; 2 Department of Gastroenterology, Epsom and St Heliers NHS Trust, Carshalton SM5 1AA, UK; 3 Department of Gastroenterology, University Hospital of Coventry and Warwickshire, Coventry CV2 2DX, UK; 4 Health, Biological & Experimental Sciences, University of Coventry, Coventry CV1 5FB, UK; 5 School of Health Sciences, University of Leicester, Leicester LE1 7RH, UK

**Keywords:** volatile organic compounds, hepatocellular cancer, pancreatic cancer, gallbladder cancer

## Abstract

**Simple Summary:**

Hepatobiliary cancers are notoriously difficult to detect, frequently leading to diagnosis in later stages of disease when curative treatment is not an option. The currently used biomarkers such as AFP (alpha-fetoprotein) and CA19.9 lack sensitivity and specificity. Hence, there is an unmet need for an alternative biomarker. Volatile organic compounds are produced by numerous tissues in the human body and subsequently excreted in breath, urine, blood, faeces, bile and saliva. Various analytical methods can be deployed to measure the concentration of these compounds in the bodily fluids. These are non-invasive and several studies have shown high patient acceptability. In light of this, we systematically reviewed the evidence available so far on the usefulness of volatile organic compounds in the detection of hepatobiliary and pancreatic cancers. Our review confirms that volatile organic compounds can be used, either alone or in combination with other biomarkers for the early diagnosis of hepatobiliary and pancreatic cancers.

**Abstract:**

Background: Hepatobiliary cancers are notoriously difficult to detect, frequently leading to diagnosis in later stages of disease when curative treatment is not an option. The currently used biomarkers such as AFP (alpha-fetoprotein) and CA19.9 lack sensitivity and specificity. Hence, there is an unmet need for an alternative biomarker. Aim: To evaluate the diagnostic accuracy of volatile organic compounds (VOCs) for the detection of hepatobiliary and pancreatic cancers. Methods: A systematic review of VOCs’ use in the detection of hepatobiliary and pancreatic cancers was performed. A meta-analysis was performed using the software R. Heterogeneity was explored through meta-regression analysis. Results: A total of 18 studies looking at 2296 patients were evaluated. Pooled sensitivity and specificity of VOCs for the detection of hepatobiliary and pancreatic cancer were 0.79 (95% CI, 0.72−0.85) and 0.81 (97.5% CI, 0.76−0.85), respectively. The area under the curve was 0.86. Meta-regression analysis showed that the sample media used contributed to heterogeneity. Bile-based VOCs showed the highest precision values, although urine and breath are preferred for their feasibility. Conclusions: Volatile organic compounds have the potential to be used as an adjunct tool to aid in the early diagnosis of hepatobiliary cancers.

## 1. Introduction

Volatile organic compounds (VOCs) are carbon-based molecules which have high vapor pressure at room temperature and are therefore in a gaseous state. Volatile organic compounds are emitted from the human body and can be measured in different biological mediums such as skin, blood, urine, bile, and breath to reflect metabolic state. Although technically any biological sample from the body may produce volatile organic compounds, some are more appealing to clinical practice. For example, urine is cheap and simple to obtain in clinical practice and contains biomarkers relevant to cancer; therefore, it has potential in screening for cancers. This has already been tested in colorectal cancer and has shown promising results [1]. In different cases of disease, the natural metabolism of the cells involved changes, resulting in a different volatile organic compound profile. Thus, the ability to detect disease-specific volatile organic compound profiles provides a novel pathway in the development of non-invasive diagnostic tests [2].

In 1971, the volatile organic compound profile of breath and urine was first characterised using gas-liquid partition chromatography to reveal 250 and 280 metabolites, respectively [3]. Since then, there have been many advances in techniques for analysing volatile organic compounds such as selected ion flow tube mass spectrometry (SIFT-MS), gas chromatography and mass spectrometry (GC/MS), gas chromatography- ion mobility spectrometry (GC/IMS), gas chromatography/time-of-flight mass (GC/TOF/MS) and electronic nose (e-nose) [4]. These techniques now allow us to analyse volatile organic compounds from many different biological mediums, providing us with the opportunity to assess the role of volatile organic compounds in clinical diagnosis.

Volatile organic compounds originate via many different mechanisms in the body. Metabolism within cells results in the release of these compounds. Hence, they are present in all humans. A total of 2746 volatile organic compounds have been detected in the healthy human body [5]. Tumour development is closely related to altered tissue metabolism due to oxidative stress [6]. This results in a change in overall VOC profile (volatilome) produced by that particular tissue. Several studies have attempted to identify the specific volatile organic compounds emitted by cancer cells [7,8,9,10,11,12,13,14,15,16]. These can be largely grouped under hydrocarbons, alcohols, aldehydes, aromatic compounds, ketones, acids, esters, ethers, and others [17]. It is noteworthy that many of these compounds may also be released in inflammatory conditions and other benign disease, so target compounds need to be specific as well as sensitive [18,19,20]. These VOCs are then exchanged into different bodily fluids, such as breath, urine, and faeces, where they can be sampled and measured. Gas chromatography-based techniques are the ‘gold standard’ at present, and they offer the benefit of the separation and identification of individual components. The newer electronic nose (e-nose) technologies are based on pattern recognition rather than detecting individual compounds and are emerging as cost effective and practically feasible methods.

Hepatocellular carcinoma is the sixth most common cause of cancer-related deaths in the western world, with an average 5-year survival rate of <15%. Within East-Asia and Sub-Saharan Africa, hepatocellular carcinoma is the second most common cause of cancer-related death due to a higher prevalence of associated risk factors [21]. Alpha-fetoproteins combined with ultrasound scans are the current tools used to screen for hepatocellular carcinoma, and 30−50% of patients do not express alpha-fetoprotein; therefore, there is a need for a new detection method [22]. Hepatocellular carcinoma is strongly linked to cirrhosis, and so it is important that a potential biomarker is also able to differentiate hepatocellular carcinoma from cases of chronic liver disease and cirrhosis [23]. Hepatocellular carcinoma detected at earlier stages will lead to improved prognosis; therefore, a potential biomarker should also be able to detect early disease [24].

Pancreatic cancer is now the third leading cause of death in men and women combined, and therefore has been included in this study due to its close relationship with the hepatobiliary system and particularly poor prognosis [25]. In most cases, diagnosis of pancreatic cancer occurs in advanced, incurable stages. Currently CA19.9 is the only biomarker used clinically in the management of pancreatic cancer; however, this is not suitable for use in early detection, as 35% of patients with respectable adenocarcinoma do not show elevated CA19.9 [26]. Chronic pancreatitis is one of the biggest risk factors for developing pancreatic cancer, and so, as with hepatocellular carcinoma, it is vital to be able to distinguish the two. They do in fact have different biomarker signatures, making them a good candidate for discrimination by volatile organic compounds [27].

The incidence of cholangiocarcinoma is rising, and is also often detected at later stages of disease where there is more opportunity for metastatic progression. Metastatic cholangiocarcinoma is particularly aggressive with a 2% 5-year survival rate [28,29]. Its detectability by volatile organic compounds has not been studied until recently [30].

Gallbladder cancer is an aggressive disease, and one of the most common cancers of the biliary tract. It is notoriously hard to diagnose, leading to poor prognosis and a 5-year survival rate of less than 5%. There is not yet a suitable biomarker for the detection of gallbladder cancer, and this leads to only 15% of patients being suitable for curative resection at the point of diagnosis [31,32]. There is an urgency to find an appropriate way to detect gallbladder cancer early, and volatile organic compounds may be the key to this.

Hepatobiliary cancers all share common difficulties within their diagnostic pathway. Diagnosis can be time consuming, invasive, and often happens at later stages of the disease, due to presenting with non-specific symptoms [33,34,35]. Survival rates of liver and pancreatic cancer are among the lowest, and it is therefore crucial to develop early detection methods to improve prognosis [25].

This review includes a systematic search of relevant hepatobiliary cancers such as hepatocellular carcinoma, cholangiocarcinoma, gall bladder cancer, and pancreatic cancer. The main objectives of this review are to determine whether volatile organic compound analysis can detect hepatobiliary cancers with sufficient accuracy to be used in diagnosis.

## 2. Methods

This systematic review has been conducted according to the Preferred Reporting Items for Systematic Reviews and Meta-analysis (PRISMA) guidelines.

### 2.1. Search Strategy

Literature search strategies were developed using medical subject headings (MeSH) and text words related to the title. The search was performed using Medline, Ovid, EMBASE, Scopus, and Cochrane with various combinations of keywords and subject headings: “volatile organic compounds”, “neoplasms”, “liver”, “cholangio”, “hepatic”, “biliary”, “pancreas”, and “pancreatic duct”. The following free words were also used in combination to ensure a maximum capture: ‘ion mobility spectrometry’, ‘gas chromatography mass spectrometry (GC/MS)’, ‘field asymmetric ion mobility spectrometry (FAIMS)’, ‘selected ion flow tube mass spectrometry (SIFT/MS)’, ‘electronic nose (e-nose)’, ‘volatilome’, ‘volatolome’ and ‘metobolome’. Reference lists of included manuscripts were also checked for additional studies. All of the articles published up to and including the 30 November 2022 were considered for this review. Details of the search strategy can be found in Supplementary files.

### 2.2. Selection of Studies

Papers retrieved from the search were imported into EndNote and duplicates were removed [36]. Papers were screened against the inclusion criteria by two independent reviewers. Disagreements on studies included were discussed and resolved together. Inclusion criteria were: (1) studies including adults only (aged ≥18), (2) clinical trials, case control studies, prospective, retrospective cohort studies, and nested case control studies, (3) studies that led to a diagnosis of a relevant cancer, and (4) studies that had a control group. Exclusion criteria were: (1) insufficient reported details to calculate true positive, true negative, false positive, and false negative, and (2) studies which were published as abstracts or reviews.

### 2.3. Data Extraction and Quality Assessment

Data was extracted by two independent reviewers. Data extracted includes authors, year of publication, cancer type, analysis technology and the sample medium used, number of cases/controls, sensitivity, specificity, true positive, true negative, false positive, and false negative. Quality was assessed using the Quality Assessment of Diagnostic Accuracy Studies-2 (QUADAS-2) tool [37]. Results from the QUADAS-2 can be found in Table S1.

### 2.4. Statistical Analysis

Statistical analysis was performed using R [38]. Where the studies did not report true positive, true negative, false positive, and false negative explicitly, they were calculated from sensitivity and specificity and their corresponding confidence intervals, or the cases used to compute them. Publication bias was assessed using a funnel plot and Deeks’ regression test for asymmetry. Bivariate meta-analysis for sensitivity and specificity was performed using the package “mada” in R [39]. Forest plots were used to summarise the results from the bivariate meta-analysis. The confidence region for sensitivity and false positive rate (1-specificity) and summary receiver operator curves (SROCs) were also produced. A bivariate meta-regression analysis was performed to determine whether the VOC media, analytical methods used, and the cancer type contributed to the heterogeneity, and also had an effect on the diagnostic performance of VOCs. If a covariate was found to be significant, a subgroup analysis was performed to further explore this. For demonstrative purposes, gallbladder cancer is grouped under cholangio carcinoma.

## 3. Results

### 3.1. Basic Characteristics of the Included Studies

**Figure 1:**
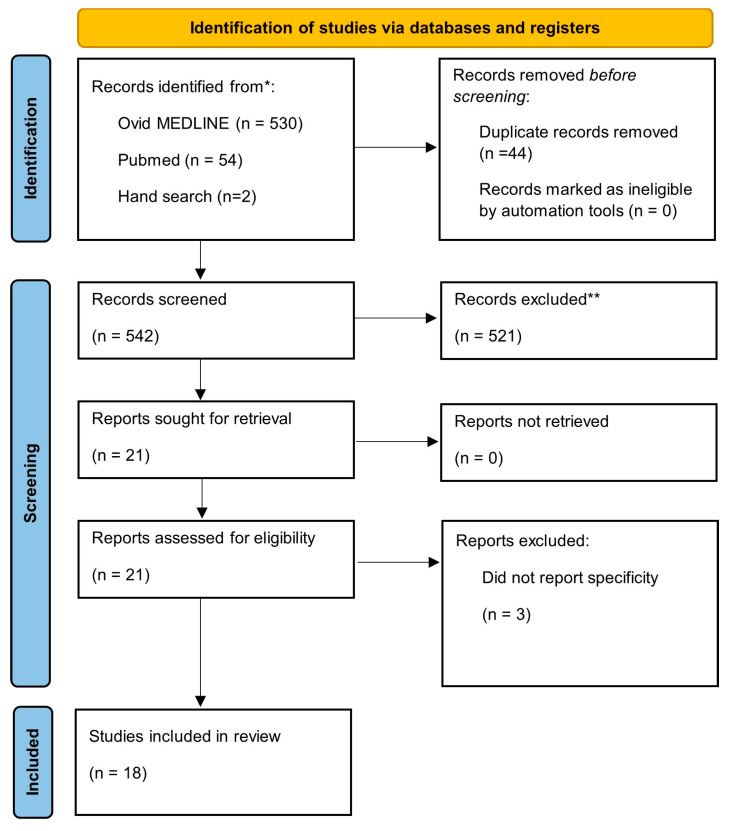
PRISMA flow diagram illustrating the study selection process. * All of the records identified from initial literature search; ** not relevant or did not have a control group or commentaries or abstract publication only.

A total of eighteen studies were included in the meta-analysis which yielded 27 study cohorts and 2296 number of patients. The study selection process is shown in the PRISMA flow diagram (Figure 1). Overall, our review includes 15 study cohorts on pancreatic cancer, 5 on hepatocellular carcinoma, 6 on cholangiocarcinoma, and 1 on both pancreatic and cholangiocarcinoma. Samples analysed across all the study cohorts included: urine, breath, blood, and bile. The basic study characteristics are summarised in Table 1.

### 3.2. Quality Assessment

Risk of bias was assessed under four domains—patient selection, index test, reference standard, and patient flow and timing—and then graded as ‘low risk’, ‘unclear’, or ‘high risk’. The results are summarised in Table S1. The greatest risk of bias was identified in patient selection and flow and timing. This was mainly due to not including a positive control group (benign disease control) or not having all patients included in the final analysis.

The funnel plot for publication bias is given in Figure S1. Deeks’ regression test for funnel plot asymmetry showed an absence of publication bias amongst the studies included in the meta-analysis (*p* = 0.97).

### 3.3. Diagnostic Accuracy of VOCs for the Detection of Hepatobiliary and Pancreatic Cancer

**Figure 2:**
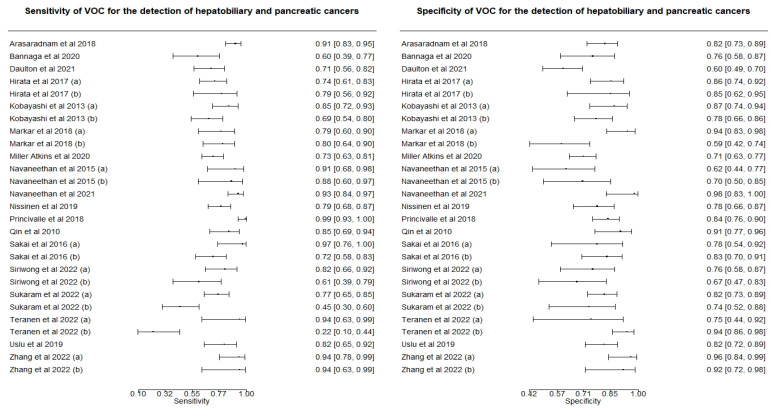
Forest plot illustrating the performance of volatile organic compounds for the detection of hepatobiliary and pancreatic cancer [30,40,41,42,43,44,45,46,47,48,49,50,51,52,53,54,55,56,57].

**Figure 3:**
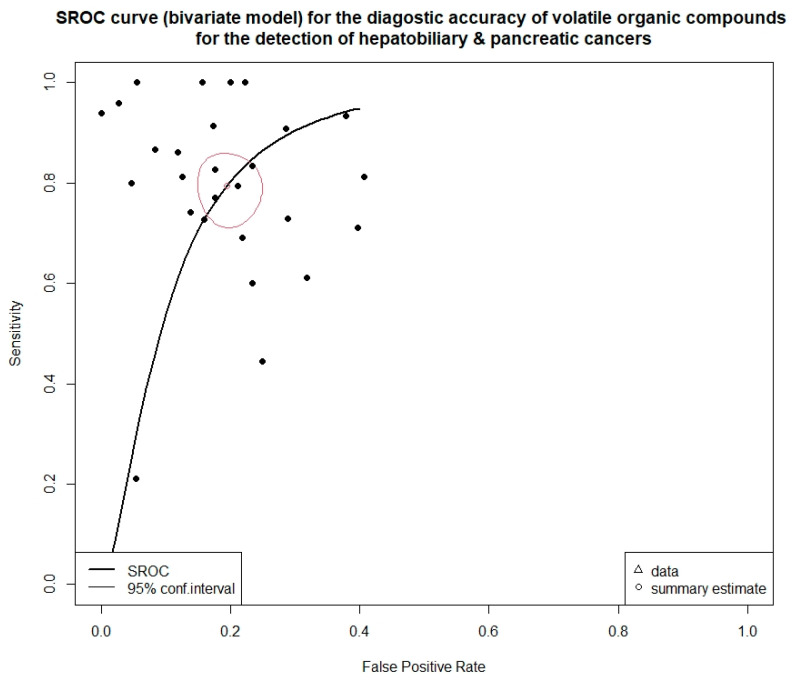
Summary receiver operator curve characteristics for volatile organic compounds in the detection of hepatobiliary and pancreatic cancer. The circle represents the confidence interval, and the dots represent the distribution of studies.

The pooled sensitivity and specificity of VOCs for the detection of any hepatobiliary and pancreatic cancer (pancreatic, hepatocellular carcinoma, cholangiocarcinoma, and gallbladder) was 0.79 (95% CI, 0.72–0.85) and 0.81 (97.5% CI, 0.76−0.85), respectively. The forest plot for the sensitivity and specificity of VOCs is given as Figure 2. Figure 3 represents the SROC curve analysis for VOCs. The area under the curve (AUC) was 0.86.

The test of heterogeneity suggests the presence of significant heterogeneity amongst the included studies. A meta-regression analysis was performed to explore the heterogeneity further, and to identify the covariates which could have influenced the performance of VOCs. The covariates included in the bivariate meta-regression analysis were VOC sample media, the method of VOC analysis, and the cancer type. The covariate sample media had four categories: breath, bile, blood, and urine. The categories for analytical methods were GC/MS, SIFT/MS, GC/IMS and, and e-nose. The cancer types included were hepatocellular, pancreatic, cholangio, and pancreatic. Only the sample media reached the statistical significance (*p* < 0.05) in meta-regression analysis (see Table S2). Hence, a subgroup analysis was performed on the sample media used.

### 3.4. Subgroup Analysis for the Sample Medium Used

**Figure 4:**
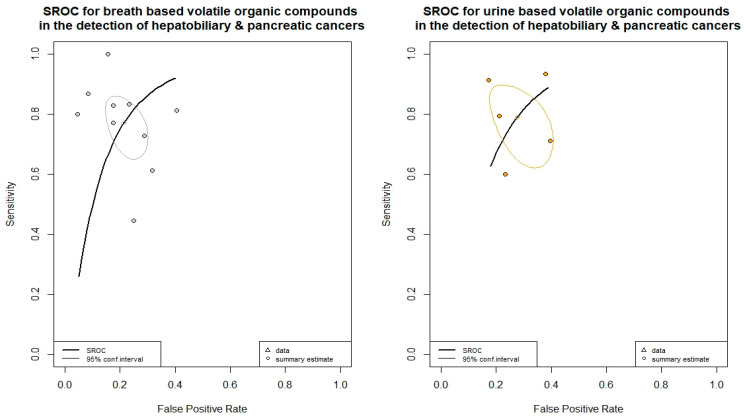
Summary receiver operator curve characteristics for breath and urine-based volatile organic compounds in the detection of hepatobiliary and pancreatic cancer. The circles represent the confidence interval, and the dots represent the distribution of studies.

**Figure 5:**
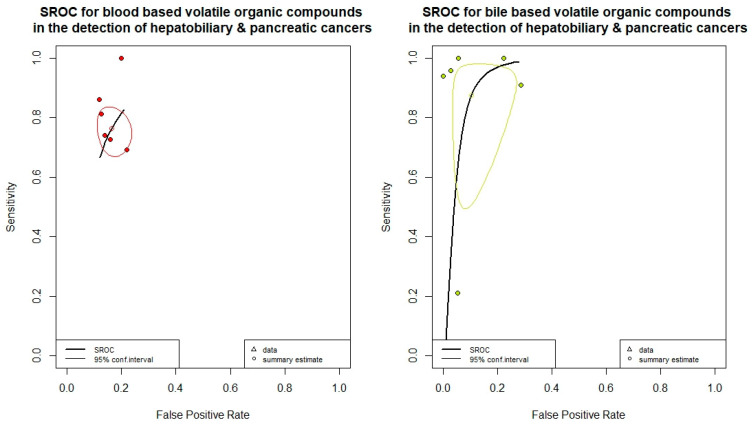
Summary receiver operator curve characteristics for blood and bile-based volatile organic compounds in the detection of hepatobiliary and pancreatic cancer. The circles represent the confidence interval, and the dots represent the distribution of studies.

A total of 7 studies (10 study cohorts) used breath as a sample medium. The sensitivity and specificity of VOCs for the detection of hepatobiliary and pancreatic cancers when breath is used as a sample medium were 0.77 (95% CI, 0.68–0.84) and 0.78 (95% CI, 0.72–0.84), respectively. The AUC was 0.84. (Figure S2, Figure 4). Five study cohorts utilised urine as their sample medium. When urine was used as a sample medium, the sensitivity was 0.79 (95% CI, 0.66–0.88) and the specificity was 0.72 (95% CI, 0.62–0.81). The AUC on the SROC was 0.81 (Figure S3, Figure 4). Blood was used as a sample medium in 6 study cohorts. The sensitivity and specificity of VOCs when blood was used were 0.76 (95% CI, 0.69–0.82) and 0.83 (95% CI, 0.78–0.88), respectively. The AUC was 0.87 (Figure S4, Figure 5). Similarly, the sensitivity for bile-based VOCs was 0.87 (95% CI, 0.59–0.97) and the specificity was 0.90 (95% CI, 0.77–0.95). The AUC under SROC was 0.94 (Figure S5, Figure 5).

## 4. Discussion

Serum biomarkers are used to help identify patients early, such as alpha-fetoprotein for hepatocellular carcinoma or CA19.9 for pancreatic cancer; however, these are not sensitive or specific enough to be a reliable diagnostic test [26,58]. There is a need for new biomarkers which could aid in the early diagnosis of hepatobiliary cancers. The results of this study showed that the VOC can be used, alone or in combination with other biomarkers, for the detection of hepatobiliary and pancreatic cancers. Importantly, its high specificity would cast some insights into its role as a screening modality.

Our study demonstrated that the cancer type did not have any effect on the performance of VOCs. ‘Oxidative stress’ has been well described as the underlying mechanism for hepatobiliary and pancreatic cancer pathogenesis [59,60,61,62]. VOCs are metabolic by-products of bacterial dysbiosis related to a specific disease state [1]. Further, the produced VOCs readily reach the liver through portal circulation and undergo extensive hepatic metabolism. Thus, the liver acts as a ‘moderator’. The common mechanistic origin of VOCs and the extensive first-pass hepatic metabolism may explain its steady performance in the spectrum of hepatobiliary and pancreatic cancers. The commonly reported eight VOCs in the included studies are presented below as Table 2.

Interestingly, we found that the analytical technique used to detect VOCs did not have any effect on its performance. This is in contrary to our previous work on colorectal cancer [63]. This finding may be due to the fact that most of the studies included in this present study used gas chromatography-based techniques.

Sample types in this review include breath, alveolar air, bile, blood, serum, and urine. Out of these, urine and breath are the least invasive. Urine samples are arguably the easiest to obtain, as they are already taken every day in clinical practice and do not require specialist equipment. Moreover, urine VOCs undergo a process of ‘biological pre-concentration’ in kidneys before excretion by separating large redundant chemicals. This process helps to identify the key ‘volatilomic signature’. A standard protocol for handling urine during volatile organic compound analysis has been published [64,65]. The volatile organic compound profile of breath has been well characterised, and there is a standard protocol available for the measurement of nitric oxide, which has the potential to be measured in real time [66,67,68]. Blood and serum are marginally more invasive, but are safe and well tolerated by most patients. Bile can only be obtained through an invasive procedure, such as ERCP or the percutaneous approach. As bile is in direct contact with these organs, the metabolic products of bacterial dysbiosis would be at their highest concentrations [69]. This is further supported by a previous study, reporting higher concentration of intramural bacteria in pancreatic cancer patients who had biliary obstructions and subsequently biliary decompression [70]. An added advantage over other matrices is that the bile VOC concentration is less affected by external factors [43]. For the aforementioned reasons, bile-based VOCs showed the highest performance indices in our subgroup analysis. It is noteworthy that the subgroups in our review had a small number of studies.

There are several factors which could have influenced the outcome of our study. Firstly, most of the studies were observational in nature and had a limited sample size. The pilot studies were also included in our review. Further, the recruitment process of the participants was not well described. These could have introduced a ‘selection’ bias. Secondly, the stage of the cancer was not well described in those included studies. Thirdly, the heterogeneity was largely due to the sample media used. Although this was further explored through subgroup analysis, the number of studies under each subgroup was small. Hence, the results should be interpreted with caution.

There are several points within the cancer pathway where volatile organic compound analysis may fit in. A quick, non-invasive, and efficient test lends itself well in primary care as an early screening tool, and may help inform decisions about referral to secondary care. This would have a similar role to the current faecal immunochemical test; however, it may appeal to more patients due to ease of sample collection. Additionally, volatile organic compounds may be used to monitor cancer progression after treatment, which has already been shown in colorectal cancer [71]. In the future, studies that evaluate the prognostic or predictive value of certain VOCs may aid in our understanding of disease progression and have a role in preventative medicine.

## 5. Conclusions

Hepatobiliary and pancreatic cancers remain one of the leading causes of death worldwide. Hence, there is an urgent need for timely detection and treatment. Currently available biomarkers, serum alpha-fetoprotein and CA 19.9, lack sensitivity and specificity on this regard.

Volatile organic compounds emerged as an attractive biomarker over the recent years. They proved to have high patient acceptability and feasibility. For the detection of hepatobiliary and pancreatic cancers, VOCs showed a sensitivity of 0.79 and a specificity of 0.81. These results are highly promising and may provide a solution for early cancer detection and treatment. Volatile organic compounds could be used alone or in combination with other biomarkers. Further, the lack of variability in their performance for the detection of different types of hepatobiliary and pancreatic cancers may cast some new insights into their complex mechanistic origin.

## Figures and Tables

**Table 1:** Basic characteristics of the studies for volatile organic compounds in the detection of hepatobiliary and pancreatic cancer and their true positive (TP), true negative (TN), false positive (FP), and false negative (FN) values.

Study name	Method	Medium	Cases	Controls	TP	FP	TN	FN	SN	SP	Type
**Bile-based**											
Navaneethan et al. 2015 (b) [40]	SIFT/MS	Bile	11	21	10	6	15	1	0.91	0.73	Cholangio
Navaneethan et al. 2021 [41]	SIFT/MS	Bile	65	23	61	0	23	4	0.94	1.00	Pancreatic
Teranen et al. 2022 (a) [42]	GC/IMS	Bile	8	9	8	2	7	0	1.00	0.78	Pancreatic
Teranen et al. 2022 (b) [42]	GC/IMS	Bile	19	75	4	4	71	15	0.21	0.95	Cholangio
Zhang et al. 2022 (a) [43]	GC/MS	Bile	24	36	23	1	35	1	0.96	0.97	Cholangio
Zhang et al. 2022 (b) [43]	GC/MS	Bile	8	18	8	1	17	0	1.00	0.94	Cholangio
**Blood-based**											
Hirata et al. 2017 (a) [44]	GC/MS	Blood	54	58	40	8	50	14	0.74	0.86	Pancreatic
Hirata et al. 2017 (b) [44]	GC/MS	Blood	16	16	13	2	14	3	0.81	0.88	Pancreatic
Kobayashi et al. 2013 (a) [45]	GC/MS	Blood	43	42	37	5	37	6	0.86	0.88	Pancreatic
Kobayashi et al. 2013 (b) [45]	GC/MS	Blood	42	64	29	14	50	13	0.69	0.78	Pancreatic
Sakai et al. 2016 (a) [46]	GC/MS	Blood	15	15	15	3	12	0	1.00	0.80	Pancreatic
Sakai et al. 2016 (b) [46]	GC/MS	Blood	44	44	32	7	37	12	0.73	0.84	Pancreatic
**Breath-based**											
Markar et al. 2018 (a) [47]	GC/MS	Breath	25	43	20	2	41	5	0.80	0.95	Pancreatic
Markar et al. 2018 (b) [47]	GC/MS	Breath	32	32	26	13	19	6	0.81	0.58	Pancreatic
Miller Atkins et al. 2020 [48]	SIFT/MS	Breath	92	159	67	46	113	25	0.73	0.71	HCC
Princivalle et al. 2018 [49]	E-nose	Breath	65	102	65	16	86	0	1.00	0.84	Pancreatic
Qin et al. 2010 [50]	GC/MS	Breath	30	36	26	3	33	4	0.87	0.92	HCC
Siriwong et al. 2022 (a) [30]	GC/IMS	Breath	30	30	25	7	23	5	0.82	0.76	Cholangio
Siriwong et al. 2022 (b) [30]	GC/IMS	Breath	18	22	11	7	15	7	0.59	0.67	Cholangio
Sukaram et al. 2022 (a) [51]	GC/MS	Breath	61	91	47	16	75	14	0.77	0.83	HCC
Sukaram et al. 2022 (b) [51]	GC/MS	Breath	36	20	16	5	15	20	0.44	0.75	HCC
Uslu et al. 2019 [52]	E-nose	Breath	29	74	24	13	61	5	0.83	0.82	Pancreatic
**Urine-based**											
Arasaradnam et al. 2018 [53]	GC/IMS	Urine	81	81	74	14	67	7	0.91	0.83	Pancreatic
Bannaga et al. 2020 [54]	GC/IMS	Urine	20	30	12	7	23	8	0.60	0.74	HCC
Daulton et al. 2021 [55]	GC/IMS	Urine	45	78	32	31	47	13	0.72	0.62	Pancreatic
Navaneethan et al. 2015 (a) [56]	SIFT/MS	Urine	15	29	14	11	18	1	0.93	0.62	Cholangio and pancreatic
Nissinen et al. 2019 [57]	FAIMS	Urine	68	52	54	11	41	14	0.79	0.79	Pancreatic

SN, sensitivity; SP, specificity; TP, true positive; TN, true negative; FP, false positive; FN, false negative.

**Table 2:** A list of frequently identified eight volatile organic compounds in patients with hepatobiliary and pancreatic cancer.

Volatile Organic Compound	Sample Medium
Acetone	Bile, breath
Benzene	Bile, breath
Acetaldehyde	Bile, breath
Valine	Blood
2-Aminoethanol	Blood
2-Butanone	Bile, breath, urine
Pentane	Bile, breath
n-Hexanone	Bile, breath, urine

## Data Availability

The data presented in this study are available on request from the corresponding author.

## Supplementary Materials

The following supporting information can be downloaded at: https://www.mdpi.com/article/10.3390/cancers15082308/s1, Figure S1: Funnel plot assessing the publication bias; Figure S2: Forest plot illustrating the performance of breath based volatile organic compounds for the detection of hepatobiliary and pancreatic cancer; Figure S3: Forest plot illustrating the performance of urine based volatile organic compounds for the detection of hepatobiliary and pancreatic cancer; Figure S4: Forest plot illustrating the performance of blood based volatile organic compounds for the detection of hepatobiliary and pancreatic cancer; Figure S5: Forest plot illustrating the performance of bile based volatile organic compounds for the detection of hepatobiliary and pancreatic cancer; Table S1: Risk of bias assessment using QUADAS-2 tool; Table S2: Regression coefficient and its p values by meta-regression analysis (fixed-effect model) for covariates sample media, analytical methods and cancer type. References [30,40,41,42,43,44,45,46,47,48,49,50,51,52,53,54,55,56,57] are cited in the supplementary materials
