# Supplementary material for: A Systematic Review and Meta-Analysis: Volatile Organic Compound Analysis in the Detection of Hepatobiliary and Pancreatic Cancers
Source: Cancers (Basel). 2023 Apr 14;15(8):2308. doi: 10.3390/cancers15082308 (PMC10136496; doi:10.3390/cancers15082308)
Supplement: Supplementary file 1 [file cancers-15-02308-s001.zip › cancers-2257526-supplementary.pdf]

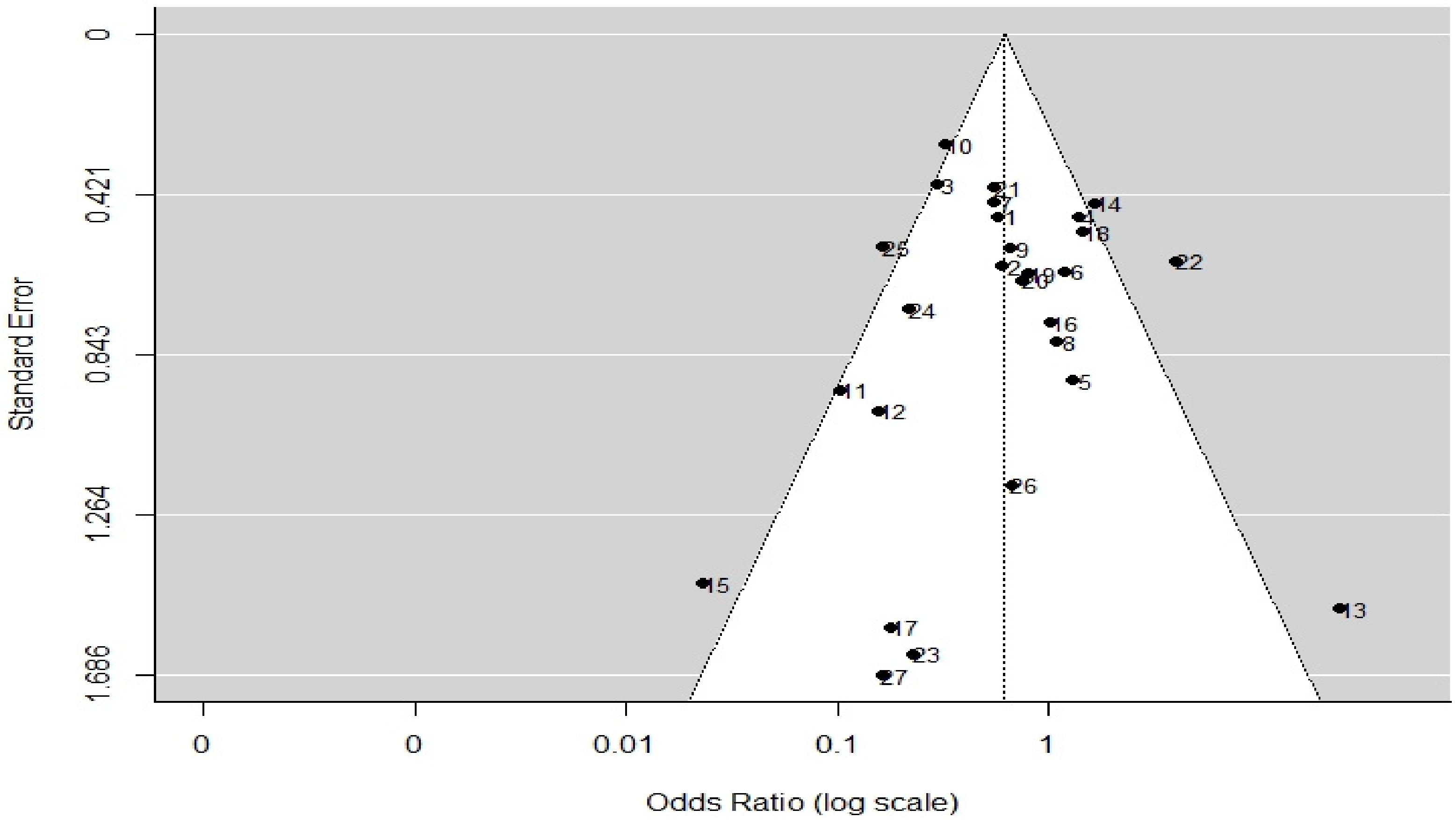

Figure S1: Funnel plot assessing the publication bias. Most of the studies are well within the 'funnel' suggest absence of publication bias. The Deeks regression test showed p = 0.97, further confirms this.

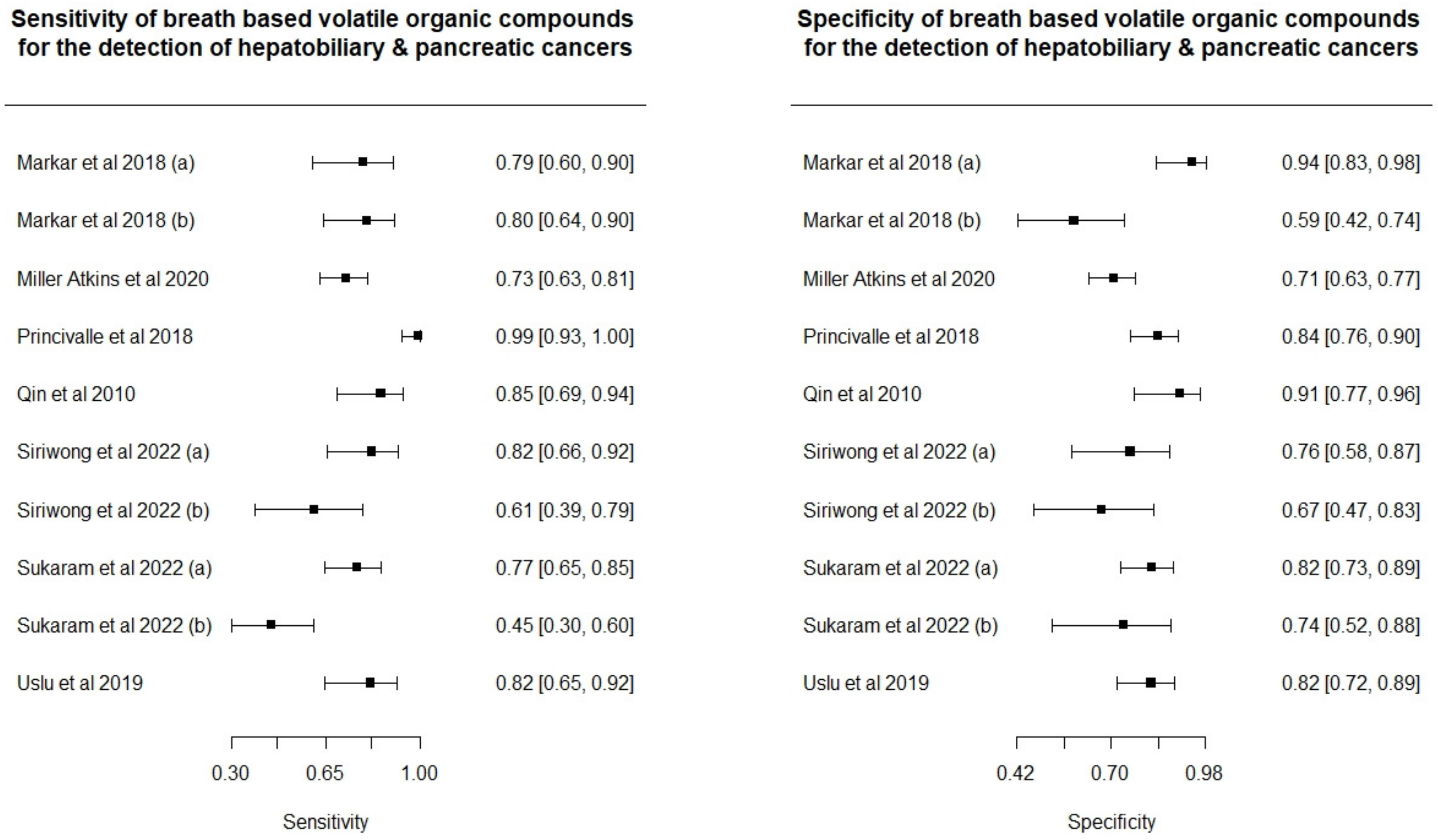

Figure S2: Forest plot illustrating the performance of breath based volatile organic compounds for the detection of hepatobiliary and pancreatic cancer [30, 47–52].

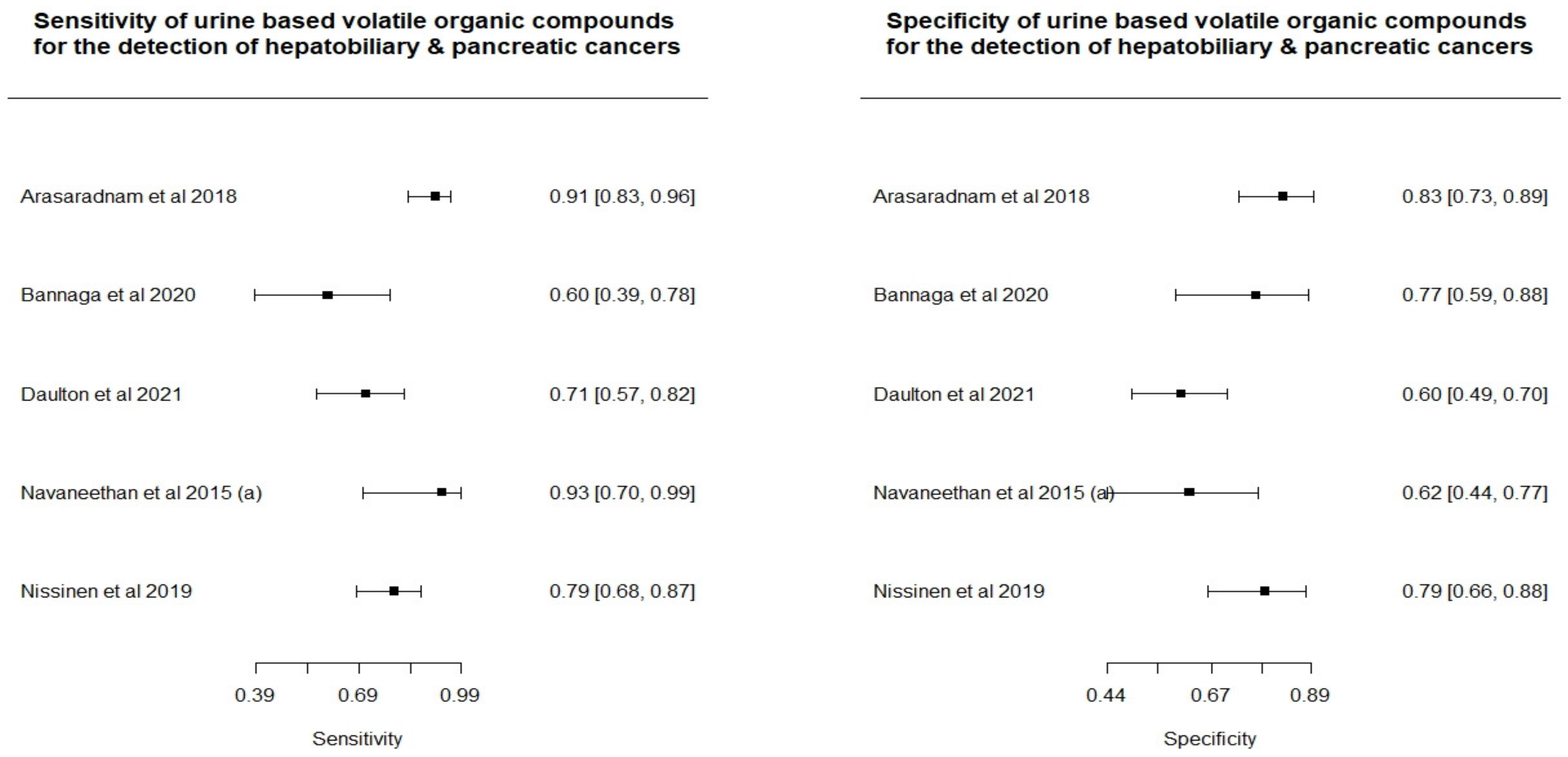

Figure S3: Forest plot illustrating the performance of urine based volatile organic compounds for the detection of hepatobiliary and pancreatic cancer [53–57].

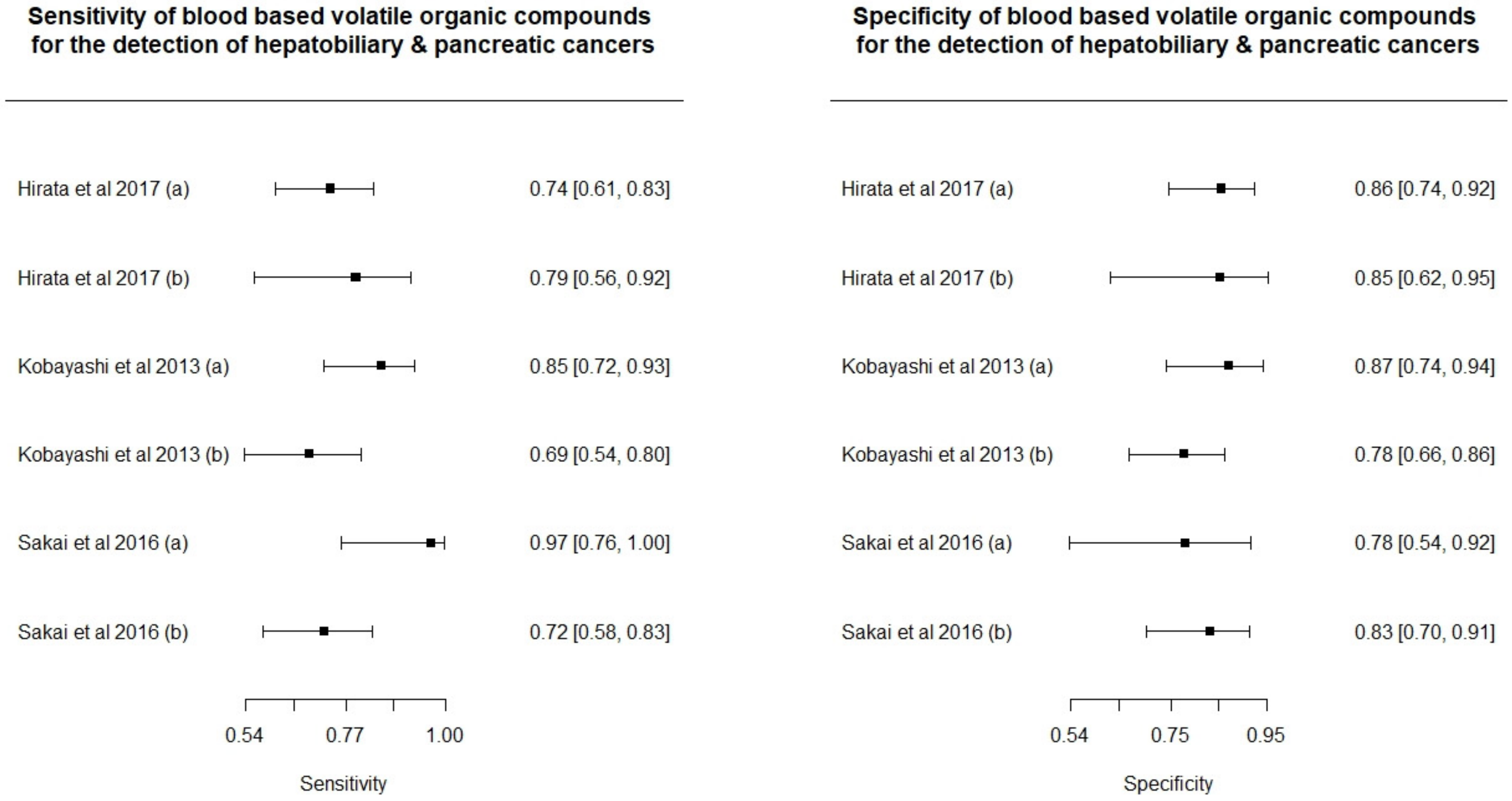

Figure S4: Forest plot illustrating the performance of blood based volatile organic compounds for the detection of hepatobiliary and pancreatic cancer [44–46].

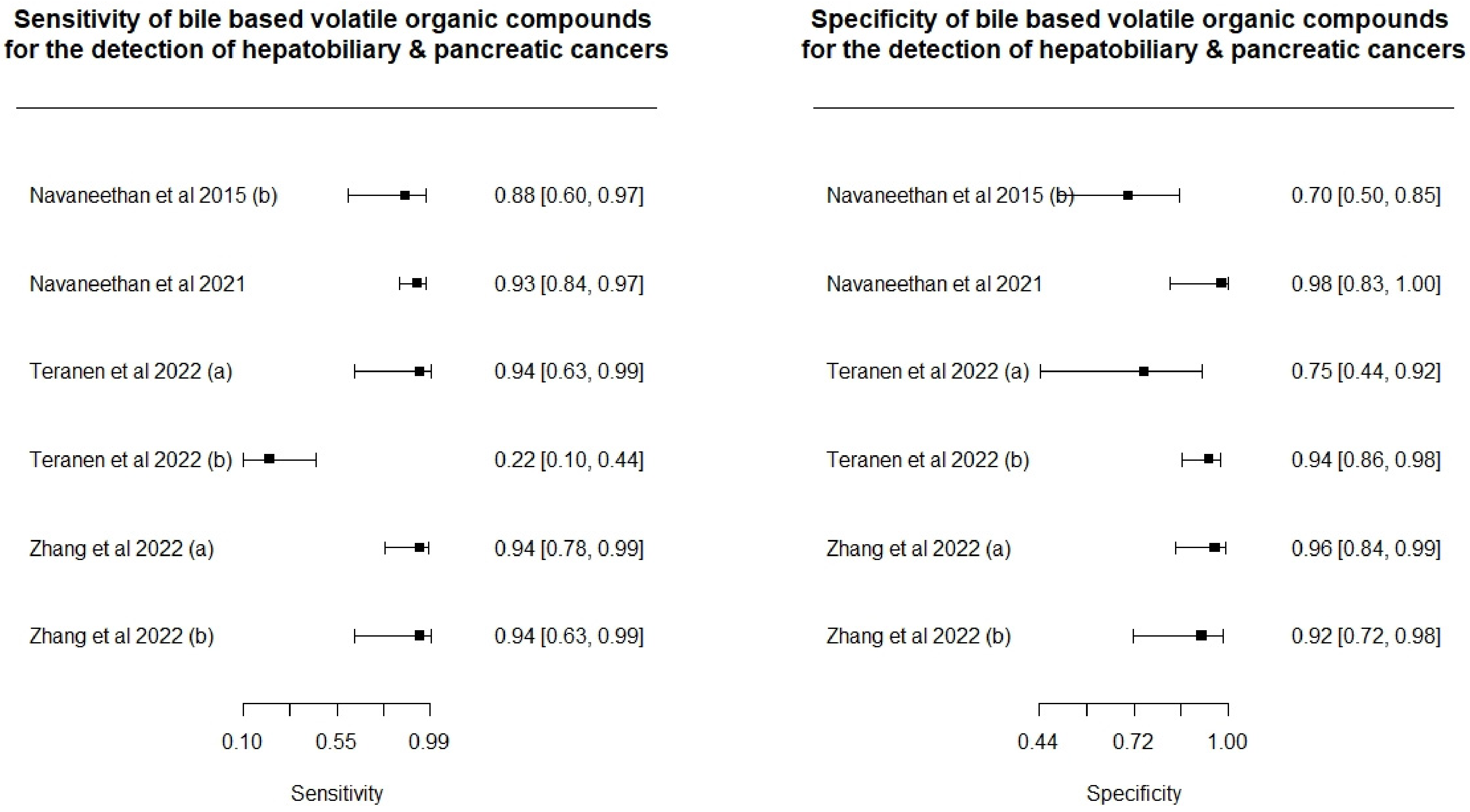

Figure S5: Forest plot illustrating the performance of bile based volatile organic compounds for the detection of hepatobiliary and pancreatic cancer 42,43,56].

Table S1: Risk of bias assessment using QUADAS-2 tool [30,40–57].

	**Risk of bias**				**Applicability concern**		
**Study**	Patient selection	Index test	Reference standard	Flow and timing	Patient selection	Index test	Reference standard
**Arasaradnam et al (2018)**	H	L	?	?	L	L	L
**Bannaga et al (2020)**	L	L	L	L	L	L	L
**Daulton et al (2021)**	L	L	?	?	L	L	L
**Hirata et al (2017)**	H	L	L	L	L	L	L
**Kobayashi et al (2013)**	H	L	L	H	L	L	L
**Markar et al (2018)**	L	L	L	L	L	L	L
**Miller Atkins et al (2020)**	L	L	L	H	L	L	L
**Navaneethan et al (2015)**	L	L	L	L	L	L	L

**Navaneethan et al (2015)**	L	L	L	L	L	L	L
**Navaneethan et al (2021)**	L	L	L	L	L	L	L
**Nissinen at al (2019)**	L	L	L	H	L	L	L
**Princivalle et al (2018)**	H	L	L	H	L	L	L
**Quin et al (2010)**	L	L	L	L	L	L	L
**Sakai et al (2016)**	H	L	L	H	L	L	L
**Sukaram et al (2022)**	L	L	L	L	L	L	L
**Uslu et al (2019)**	L	L	?	H	L	L	L
**Zhang et al (2022)**	L	L	L	L	L	L	L
**Teranen et al (2022)**	L	L	L	L	L	L	L
**Siriwong et al (2022)**	L	L	L	H	L	L	L

Table S2: Regression coefficient and its p values by meta-regression analysis (fixed-effect model) for covariates sample media, analytical methods and cancer type. For sample media – blood, breath and urine were compared to bile (intercept); for analytical methods – GC/IMS, GC/MS and SIFT/MS were compared with e-nose (intercept); for cancer types – cholangio &pancreatic, pancreatic and HCC were compared to cholangio carcinoma (intercept). P <0.05 was considered as significant.

	**Logit transformed sensitivity**		**Log it transformed false positive rate**	
	**Regression coefficient**	**p value**	**Regression coefficient**	**p value**
**Sample media**				
Intercept	1.59	0.00	-2.08	0.00
Blood	-0.32	0.60	0.49	0.24
Breath	-0.38	0.05	0.79	0.03
Urine	-0.24	0.70	1.13	0.00
**Analytical method used**				
Intercept	2.31	0.00	-1.58	0.00
GC/IMS	-1.45	0.09	0.38	0.41
GC/MS	-1.01	0.22	-0.03	0.93
SIFT/MS	-0.47	0.61	0.67	0.19
**Cancer type**				
Intercept	1.00	0.01	-1.64	0.00
Cholangio and pancreatic	1.26	0.28	1.17	0.09
HCC	-0.20	0.70	0.35	0.40
Pancreatic	0.54	0.24	0.22	0.52

Search strategy

(a) OVID MEDLINE

1	(Hepatobiliary or biliary or pancreatic or hepatocellular or gallbladder or cholangiocarcinoma or liver or cholangio or hepatic or pancreas or pancreatic duct*).mp
2	Exp Liver diseases/ or Hepatobiliary.mp or exp Liver or exp Carcinoma, Hepatocellular/ or exp Biliary tract disease/ or exp Liver neoplasms
3	(cancer* or tumour* or tumor* or malignancy* or malignant or neoplasm* or carcinoma* or adenocarcinoma*or sarcoma*).mp
4	Cancer.mp or exp Neoplasms/
5	(Volatile organic compound analysis or volatile organic compound or volatile organic compounds or VOC or Votalome or Volatilome or Volatile compounds).mp
6	Volatile organic compounds.mp or exp Volatile organic compounds/ or exp Breath tests/ or exp Gas chromatography-Mass spectrometry/
7	1 or 2
8	3 or 4
9	7 and 8
10	5 or 6
11	9 and 10

(b) PUBMED

#11	#9 AND #10
#10	#7 AND #8
#9	#5 OR #6
#8	#3 OR #4
#7	#1 OR #2
#6	"Volatile organic compounds" [mh]
#5	**"Volatile organic compound analysis"** [tw] **OR** "Volatile organic compound" [tw] OR "Volatile organic compounds" [tw] OR VOC [tw] OR Votalome [tw] OR Volatilome [tw] OR "Volatile compounds" [tw]
#4	Neoplasms [mh]
#3	Cancer* [tw] OR Tumour* [tw] OR Tumor* [tw] OR Malignancy* [tw] OR Malignant [tw] OR Neoplasm* [tw] OR Carcinoma* [tw] OR Adenocarcinoma* [tw] OR Sarcoma* [tw]
#2	Liver [mh] OR cholangio [mh] OR hepatic [mh] OR biliary [mh] OR pancreas [mh] OR "pancreatic duct*" [mh]
#1	Hepatobiliary [tw] OR biliary [tw] OR pancreatic [tw] OR hepatocellular [tw] OR gallbladder [tw] OR cholangiocarcinoma [tw]
